# Spatiotemporal dynamics, risk areas and social determinants of dengue in Northeastern Brazil, 2014–2017: an ecological study

**DOI:** 10.1186/s40249-020-00772-6

**Published:** 2020-11-03

**Authors:** Rodrigo Feliciano do Carmo, José Valter Joaquim Silva Júnior, Andre Filipe Pastor, Carlos Dornels Freire de Souza

**Affiliations:** 1 Post Graduation Program in Health and Biological Sciences, Federal University of São Francisco Valley (UNIVASF), Av. José de Sá Maniçoba, s/n, Centro, Petrolina, PE Brazil; 2 Post Graduation Program in Bioscience, Federal University of São Francisco Valley (UNIVASF), Petrolina, Brazil; 3 Virology Sector, Department of Preventive Veterinary Medicine, Federal University of Santa Maria, Camobi, Santa Maria, Brazil; 4 Department of Microbiology and Parasitology, Federal University of Santa Maria, Camobi, Santa Maria, Brazil; 5 Virology Sector, Keizo Asami Immunopathology Laboratory, Federal University of Pernambuco, Recife, Brazil; 6 Federal Institute of Education, Science and Technology of Sertão Pernambucano (IF Sertao-PE), Floresta, Brazil; 7 Department of Medicine, Federal University of Alagoas (UFAL), Arapiraca, Brazil

**Keywords:** Arboviruses, Dengue, Dengue virus, Epidemiology, GIS, Poverty

## Abstract

**Background:**

Dengue fever is an arthropod-borne viral disease caused by dengue virus (DENV) and transmitted by *Aedes* mosquitoes. The Northeast region of Brazil is characterized by having one of the highest dengue rates in the country, in addition to being considered the poorest region. Here, we aimed to identify spatial clusters with the highest dengue risk, as well as to analyze the temporal behavior of the incidence rate and the effects of social determinants on the disease transmission dynamic in Northeastern Brazil.

**Methods:**

This is an ecological study carried out with all confirmed cases of dengue in the Northeast Brazil between 2014 and 2017. Data were extracted from the National Notifiable Diseases Information System (SINAN) and the Brazilian Institute of Geography and Statistics (IBGE). Local empirical Bayesian model, Moran statistics and spatial scan statistics were applied. The association between dengue incidence rate and social determinants was tested using Moran’s bivariate correlation.

**Results:**

A total of 509 261 cases of dengue were confirmed in the Northeast during the study period, 53.41% of them were concentrated in Pernambuco and Ceará states. Spatial analysis showed a heterogeneous distribution of dengue cases in the region, with the highest rates in the east coast. Four risk clusters were observed, involving 815 municipalities (45.45%). Moreover, social indicators related to population density, education, income, housing, and social vulnerability showed a spatial correlation with the dengue incidence rate.

**Conclusions:**

This study provides information on the spatial dynamics of dengue in northeastern Brazil and its relationship with social determinants and can be used in the formulation of public health policies to reduce the impact of the disease in vulnerable populations.

## Background

Dengue is an acute arthropod-borne viral infection transmitted mainly by the *Aedes aegypti* mosquito, and is the most frequent arboviral disease globally [1]. Dengue occurs mainly in the tropics and subtropics with an estimated burden of 390 million cases per year, of which 96 million cases manifest symptomatically, 2 million cases develop severe disease, and 21 000 deaths occurs annually [1, 2]. The worldwide spread of dengue is a complex issue, which may be accelerated by several factors, such as climate change, population growth, rapid and unplanned urbanization, the movement of people for trade, tourism, or forced by natural disasters, fragilities in public health and in vector control programs [3–5].

From 2010 to 2019, more than 16 million cases of dengue were reported throughout the American continent and about 10 million cases (~ 62%) were reported only in Brazil [6]. The disease is an important public health concern in the country, with simultaneous circulation of the four viral serotypes (DENV1, DENV2, DENV3, and DENV4) [7]. Dengue has a wide geographical distribution in the country and, despite the intensification of control measures, there has been an increase in the number of severe cases, hospitalizations and deaths in recent years [8, 9]. Historically, the regions in the country with the highest both dengue incidences and fatal cases have been the Southeast, followed by the Northeast. Together, Southeast and Northeast regions have contributed 43% and 27%, respectively, of the total fatal dengue cases in Brazil [10]. The Northeast region is one of the poorest regions in Brazil, also presenting the greatest risks of hospitalization for dengue in the country [11].

The Northeast is one of the five regions of Brazil (North, Northeast, Midwest, Southeast and South). Over the years, the region has faced serious social difficulties, presenting the lowest human development index (HDI = 0.667) and the highest Gini inequality index (0.522). In addition, 61.2% of the municipalities have low HDI (< 0.600) and only 1.9% have a very high HDI (> 0.800) [12]. 43.5% of the population of Northeastern Brazil live in poverty, living on USD 5.5/day, and the illiteracy rate in people aged 15 or over reaches 14.48%, more than double the national average (6.92%) [13, 14].

Urban growth provides a great source of susceptible and infected individuals concentrated in restricted areas. This fact, associated with the precarious conditions of basic sanitation, inadequate housing, cultural and educational factors, provides ecological conditions favorable to dengue virus (DENV) transmission [15, 16]. Studies carried out in capitals of the Brazilian Northeast, Recife and Fortaleza, for example, demonstrated a greater risk of infection in socioeconomically deprived areas [17, 18].

In this context, spatiotemporal analysis can contribute to the understanding of the dynamics of the disease in target populations, as well as to the identification of risk areas, contributing to the implementation of public policies with adequate control and prevention actions [19]. Therefore, this study aimed to identify spatial clusters with the highest risks of DENV transmission, to analyze the disease transmission dynamic and to find socioeconomic factors associated with dengue occurrence in the Northeast region of Brazil.

## Methods

### Study area

**Fig. 1:**
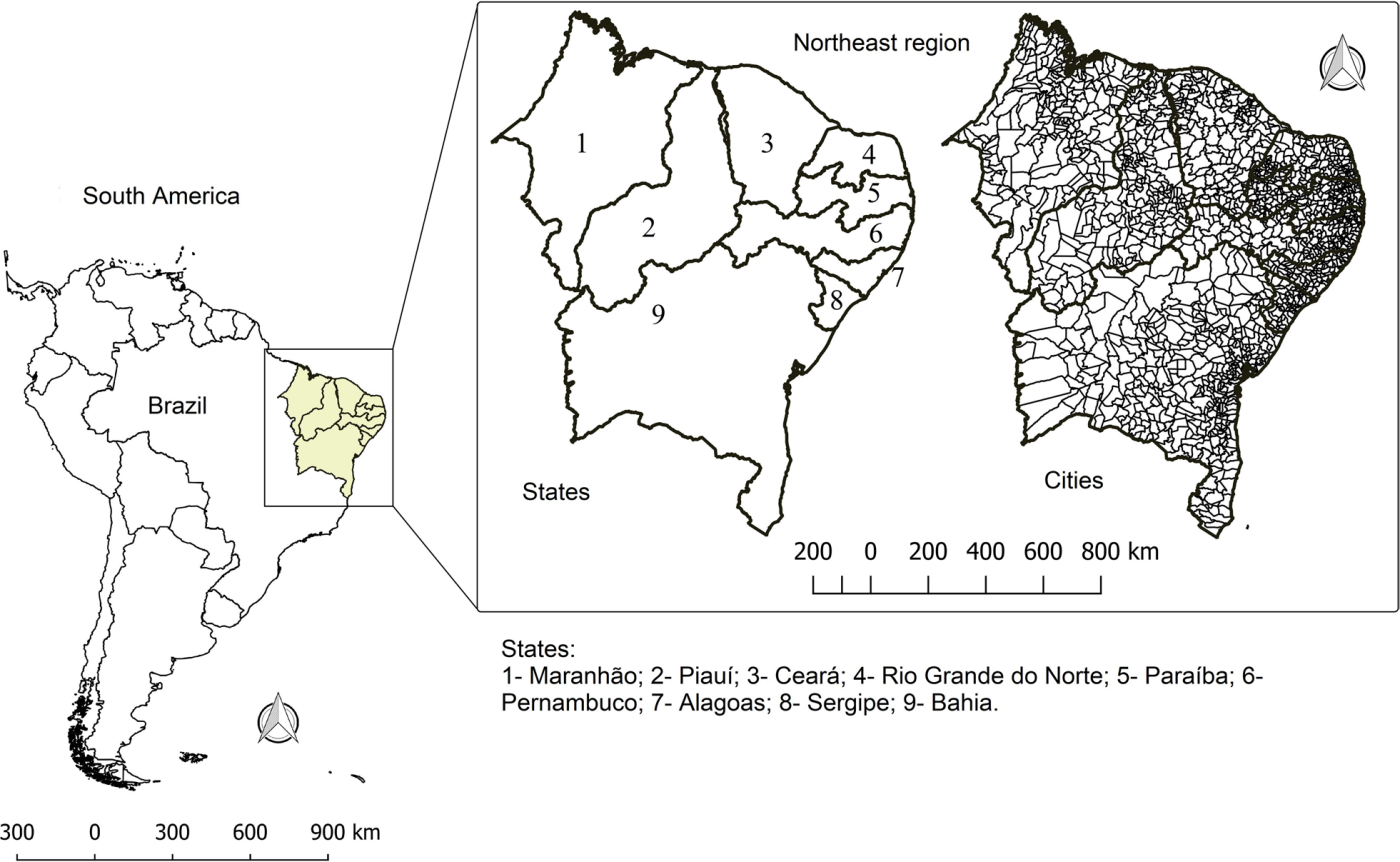
Location of the study area. Northeast, Brazil. 2020

This study was carried out in the Northeast region of Brazil, which is located between the latitudes of 1° and 18° 30′ S, and longitudes of 34° 20′ e 48° 30′ W. The Northeast region includes nine states: Maranhão (MA), Piauí (PI), Ceará (CE), Rio Grande do Norte (RN), Paraíba (PB), Pernambuco (PE), Alagoas (AL), Sergipe (SE) and Bahia (BA). It occupies a territorial area of 1 554 291 km^2^ (18% of Brazilian territory) and had an estimated population of 57.2 million inhabitants in 2017 (second largest population among Brazilian regions) (Fig. 1). Approximately 60% of the Northeast area has a semiarid climate and an average annual rainfall of 500 mm year^−1^. Additionally, the region has experienced an increase in air temperature and dryness in the last decades [20].

### Data sources

This is an ecological study, including all dengue cases confirmed and registered in residents in the Northeastern region of Brazil from 2014 to 2017. As of 2014, there was a change in the clinical classification of dengue in Brazil, which started to adopt the classification proposed by WHO (dengue, dengue with alarm signs or severe dengue) [21] and the registration started to occur in the online version of National System of Notifiable Diseases (Sinan Online). For this reason, 2014 was the initial year of this study.

The following inclusion criteria were adopted: (i) cases notified between 2014 and 2017; (ii) individuals residing in the Northeast states; and (iii) cases closed in the information system and with clinical classification. In the study, cases whose clinical classification field was ignored/blank or registered as inconclusive were excluded.

These data were extracted from the National Notifiable Diseases Information System (SINAN) [22]. The population data, necessary for calculating the incidence rate, were obtained from the Brazilian Institute of Geography and Statistics (IBGE) [23].

For the calculation of the incidence coefficient, the following equation was adopted:

$$\text{Incidence} = \frac{\mathrm{Number\,of\,confirmed\,dengue\,cases }}{\mathrm{Population\,living\,in\,the\,place\,and\,period}} \times 100 000$$

From the IBGE database, we then selected indicators that express social vulnerability and grouped them into five categories. The indicators were selected according to the social determinants previously reported for dengue, also considering the transmission characteristics of the disease and the availability of the indicators for all municipalities in the study area. These indicators were grouped according to the meaning they express.

a Population: percentage of urban population and population density (inhab/km^2^);
b Education: percentage of people aged 6 to 14 who do not attend school; Illiteracy rate of individuals aged 18 or over; percentage of people aged 18 or over without complete elementary education and in informal occupation;
c Income: average income of the employed; percentage of income from work; percentage of people aged 15 to 24 who do not study, do not work and have a per capita household income equal to or less than half the minimum wage (2010);
d Housing: percentage of households with access to piped water; percentage of households with access to electricity;
e Social vulnerability: percentage of mothers who are heads of household without elementary school and with minor children, in the total of mothers who are heads of families; percentage of vulnerable people who spend more than an hour to work among the employed population.

### Data analysis

The statistical treatment of the data was carried out in three steps:

#### Time trend analysis

Trend analysis was carried out with the use of a joinpoint regression model. Trends were classified as increasing, decreasing, or stationary. The annual percent change (APC) was calculated, considering a confidence interval of 95% and a significance of 5%.

The *joinpoint* regression model for the observations, (*x*_1_, *y*_1_),…, (*x*_*n*_, *y*_*n*_), where *x*_1_ ≤ … ≤ *x*_*n*_ represents the time variable, and *y*_*i*_, (*i* = 1, 2,…, *n*) is the response variable, may be written as:

$$\sum {y}_{i}|{x}_{i}]= {\beta }_{0}+{\beta }_{1}{x}_{1}+{\gamma }_{1}\left({x}_{i}-{\tau }_{1}\right)+\dots +{\gamma }_{n}\left({x}_{i}-{\tau }_{n}\right)$$

where β_0_, β_1_, y_1_,…, γ_n_ are regression coefficients, and y_k_, K = 1, 2,…, n, n < N, is the k-th unknown *joinpoint* where

$$\left({x}_{i}-{\tau }_{k}\right)=\left({x}_{i}-{\tau }_{k}\right)if\left({x}_{i}-{\tau }_{k}\right)>0=0, otherwise.$$

The analyses were performed using the Joinpoint Regression Program (version 4.5.0.1, National Cancer Institute, Bethesda, MD, USA) [24].

#### Spatial analysis and identification of risk areas

Initially, the dengue incidence rate was carried out by the application of a local empirical Bayesian model [25]. The modeling aims to identify the posterior distribution (unobserved quantities of a given phenomenon), based on the application of Bayes' theorem, involving sample data (likelihood function), and a set of observed data (a priori distribution) [25]. The correction reduces random fluctuation caused by rare events, municipalities with small populations and underreporting of the disease.

After correction, spatial autocorrelation was calculated using the Moran global index. The global index provides a general measure of spatial association, whose expression and calculation consider a proximity matrix of order 1. The index varies between − 1 and + 1, where values equal to zero indicate the absence of spatial autocorrelation, and values close to + 1 and − 1 indicate the existence of positive or negative spatial autocorrelation, respectively [26].

Moran's global index (I Global) is given by the equation:

$$I = \frac{{\sum\limits_{i = 1}^n {\sum\nolimits_{j = 1}^n {{w_{ij}}} } \left( {{z_i} - \overline z } \right)\left( {{z_j} - \overline z } \right)}}{{\sum\nolimits_{i = 1}^n {{{\left( {{z_i} - \overline z } \right)}^2}} }}$$

where *n* is the number of areas, z_*i*_ the value of the attribute considered in area *i*, *z* is the mean value of the attribute in the region of study, and *wij*, the elements of the normalized spatial proximity matrix.

Once the global dependence was verified, the Local Index of Spatial Association (LISA) was calculated. LISA is a decomposition of the I Global, in which it is possible to elaborate an analysis of the local pattern of spatial data.

LISA can be expressed for each area *i* from the normalized *z*_*i*_ values of the attribute as:

$${I}_{i}=\frac{{z}_{i}\sum_{j=1}^{n}{w}_{ij}{z}_{j}}{\sum_{j=1}^{n}{z}_{j}^{2}}$$

Based on LISA, the municipalities are positioned in the quadrants of the Moran scattering diagram in the following manner: Q1 (high–high), municipalities where the attribute value and the average value of the neighbors are above the average of the set and which are, therefore, considered highest priority for intervention; Q2 (low–low), the attribute value and the average of the neighbors are below the average of the set; Q3 (high–low), attribute value is greater than that of neighbors and the average of neighbors is less than that of the set; and Q4 (low–high), the attribute value is less than that of the neighbors and the average of the neighbors is greater than the average of the set. The municipalities classified as high–low and low–high have intermediate priority [26].

Then, three spatial scanning analysis techniques were applied to identify high-risk clusters: purely spatial, spatiotemporal and spatial variation of the temporal trend. The Poisson discrete probability model and the maximum likelihood method were adopted, whose alternative hypothesis is that there is a high risk inside the window compared to the outside. For the identified areas, the model calculates the relative risk (RR) [27].

The scan statistic establishes a flexible circular window in the map, positioned on each of the several centroids and whose radius is established in 50% of the total population at risk. The flexibility of the window was justified by not knowing the size of the cluster a priori, since the population at risk is not geographically homogeneous. Monte Carlo simulations (999 permutations were adopted) were used to obtain *P* values, with clusters with *P*-value < 0.05 being significant.

#### Spatial correlation of dengue with social determinants

Initially, all indicators were submitted to global Moran statistics to assess the presence of spatial dependence. Then, the social indicators were subjected to bivariate spatial correlation with the raw incidence rate and smoothed rate as dependent variable. Moran's bivariate analysis allows to identify whether the value of an attribute observed in a given region is spatially related to the values of another variable observed in neighboring regions, i.e., the degree of linear spatial correlation (whether positive or negative) between the value of one variable and the average of another variable in neighboring locations [26].

Bivariate Moran’s I can be defined as:

$${I}_{zi, zii}=\frac{Zi\mathrm{w}ijZii}{ZiZii}$$

where *n* is the number of areas, *zi and zii* are the values of the attributes considered in area *i*, and *wij*, the elements of the normalized spatial proximity matrix.

The analyses were performed using the following software: Terra View (version 4.2.2, Brazilian Space Research Institute, São José dos Campos, SP, Brazil), Stat Scan (version 9.1, National Cancer Institute, Bethesda, MD, USA) and QGis (version 2.14.11 Open Source Geospatial Foundation, Beaverton, OR, USA).

### Ethics statement

The study did not require research ethics committee approval because it used public-domain aggregate secondary data and no individual patients were identifiable.

## Results

### Most dengue cases are concentrated in two states

From 2014 to 2017, 509 261 cases of dengue were confirmed in the Northeast, 53.41% (*n* = 272 070) of them appeared only in two states: Ceará (29.01%; *n* = 147 768) and Pernambuco (24.41%; *n* = 124 302). These two states ranked first and third in incidence rates (413.55/100 000 and 331.41/100 000 inhabitants, respectively). Piauí was the unique state with a decreasing trend in the period (APC = − 22.6%; *P* < 0.001) (Table 1).

**Table 1:** Number of confirmed cases, incidence rate and temporal trend of dengue in Northeastern Brazil, 2014–2017

State	Number of confirmed cases					Incidence rate/100 000						Trend	
	2014	2015	2016	2017	2014–2017	2014	2015	2016	2017	2014–2017	APC	95% CI (*P-*value)	Trend
Maranhão	1898	5524	13 052	5526	26 000	27.70	80.01	187.69	78.94	93.83	54.40	− 48.9 to 365.4 (*P* = 0.2)	Stationary
Piauí	6620	6485	3612	4127	20 844	207.22	202.40	112.45	128.20	162.46	− 22.60	− 32.4 to − 11.3 (*P* < 0.001)*	Decreasing
Ceará	19 824	58 889	42 038	27 017	147 768	224.18	661.34	468.98	299.51	413.55	2.80	− 58.7 to 156.2 (*P* = 0.9)	Stationary
Rio Grande do Norte	4442	6087	9905	1879	22 313	130.32	176.84	285.04	53.58	161.31	− 14.70	− 72.4 to 163.7 (*P* = 0.6)	Stationary
Paraíba	3935	14 099	14 691	2723	35 448	99.77	354.94	367.33	67.64	222.37	− 9.90	− 83.8 to 399.6 (*P* = 0.8)	Stationary
Pernambuco	6983	72 425	39 223	5671	124 302	75.27	775.00	416.81	59.86	331.41	− 14.70	− 93.0 to 943.3 (*P* = 0.08)	Stationary
Alagoas	11 172	22 369	14 357	2807	50 705	336.33	669.54	427.42	83.15	378.47	− 37.10	− 83.7 to 142.5 (*P* = 0.3)	Stationary
Sergipe	2074	7049	1941	353	11 417	93.44	314.28	85.67	15.43	126.62	− 50.80	− 91.3 to 179.4 (*P* = 0.2)	Stationary
Bahia	9038	27 156	30 817	3453	70 464	59.75	178.62	201.73	22.50	115.61	− 22.50	− 88.4 to 415.5 (*P* = − 0.6)	Stationary
Total	65 986	220 083	169 636	53 556	509 261	117.44	389.12	298.05	93.54	224.43	− 9.90	− 77.2 to 256.4 (*P* = 0.8)	Stationary

*APC* Annual percent change, *CI* Confidence interval

^*^Statistically significant (*P* < 0.05)

**Fig. 2:**
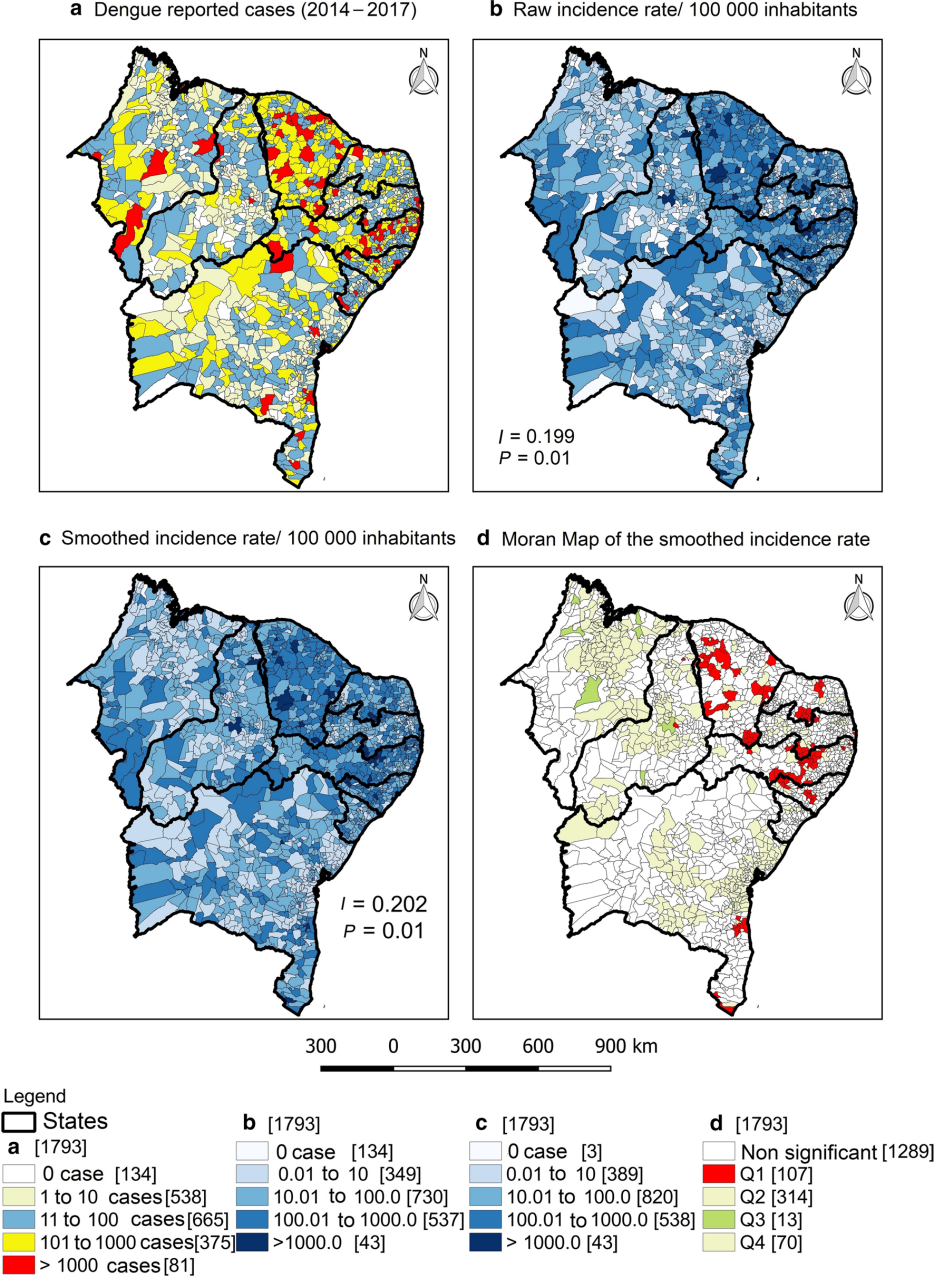
Exploratory spatial analysis of the occurrence of dengue in Northeast Brazil, 2014–2017. **a** Number of dengue reported cases, **b** raw incidence rate/100 000 inhabitants, **c** smoothed incidence rate/100 000 inhabitants and **d** Moran Map of the smoothed incidence rate. Values in brackets represent the number of municipalities. *I* = Moran’s Index

A total of 7.4% (*n* = 134) of the municipalities did not register any cases of dengue in the period, and 4.5% (*n* = 81) registered more than 1000 cases. These 81 municipalities accounted for 70.3% (*n* = 358 306) of the total number of cases in the region. Fortaleza-CE and Recife-PE occupied the first two positions in absolute number of cases and in incidence rate among the capitals, with 68 504 (65 867/100 000 inhabitants) and 38 935 (60 039/100 000 inhabitants) records, respectively (Fig. 2).

In regard to the raw rate, only 2.18% (*n* = 43) of the municipalities had an incidence greater than 1000 cases/100 000 inhabitants, highlighting the municipalities of Guamaré-RN (3750.06; *n* = 2220) and Monteiro-PB (3522.66; *n* = 4636). The smoothing by the Bayesian model reduced the random fluctuation of the data, reducing the number of silent municipalities to three (two in Maranhão state and one in Pernambuco state). On the other hand, the number of municipalities with an incidence greater than 1000/100 000 remained the same (*n* = 43). In the Moran Map, 107 municipalities were classified in the Q1 quadrant of the Moran scattering diagram (Fig. 2).

### Dengue incidence rate is higher in more populous municipalities

**Fig. 3:**
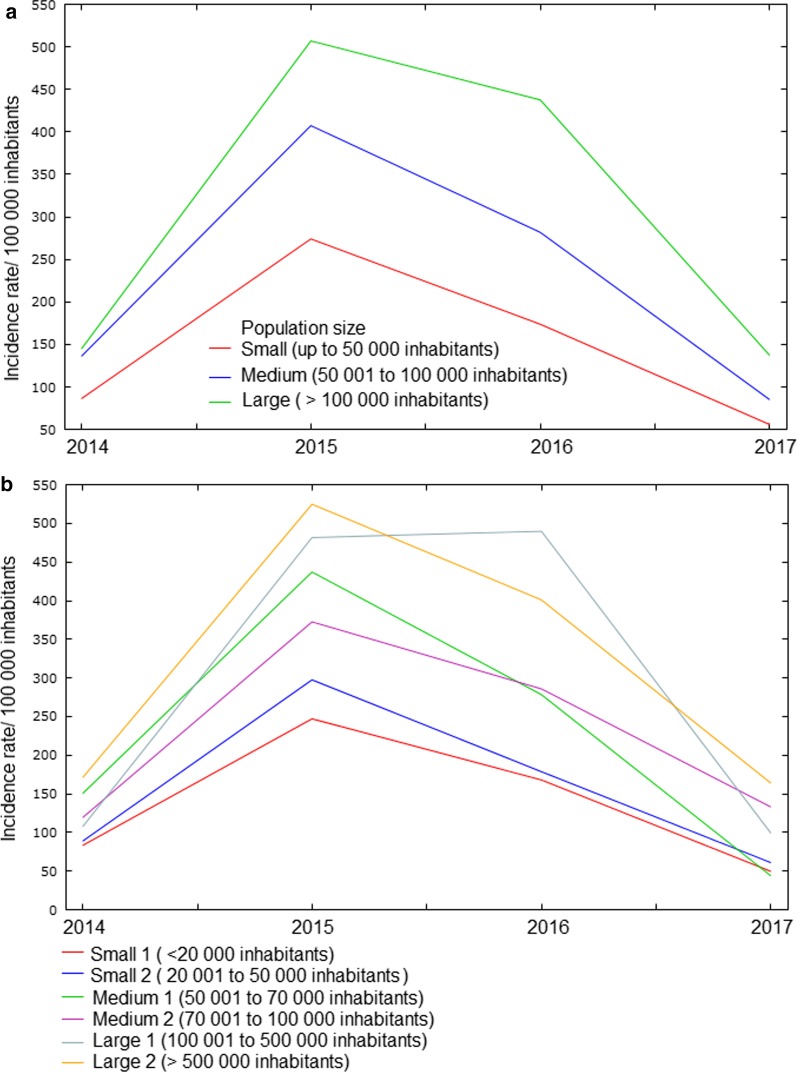
Dengue incidence rate according to the population size of municipalities in Northeast Brazil, 2014–2017

Over the four years studied, incidence rates were higher in municipalities with a larger population. In municipalities with more than 100 thousand inhabitants, the incidence rate was 1.92 times higher than that observed in municipalities with less than 50 thousand inhabitants (275.22/100 000 and 143.21/100 000, respectively). In addition, 89.7% (*n* = 1610) of the municipalities in the Northeast are small, which together accounted for 29.0% of the records (*n* = 147 935). In this group, the rates were higher in those with a population between 20 001 and 50 thousand inhabitants (159.47/100 000) (Fig. 3 and Table 2).

**Table 2:** Comparison of average dengue incidence rates according to population size in the municipalities of Northeast Brazil, 2014–2017

Population size	No. municipalities (%)	No. cases^a^ (%)	Mean (± *SD*)	Median (IQR)	*P-*value
Small (up to 50 000)	1610 (89.7)	147 935 (29.0)	143.21 ± 331.50	33.97 (122.50)	< 0.001^b^
Small 1 (up to 20 000)	1157 (64.5)	63 831 (12.5)	136.84 ± 328.52	29.79 (109.40)	
Small 2 (20 001 to 50 000)	453 (25.2)	84 104 (16.5)	159.47 ± 338.83	46.22 (145.73)	
Medium (50 001 to 100 000)	122 (6.8)	75 246 (14.8)	229.25 ± 302.78	113.91 (307.69)	
Medium 1 (50 001 to 70 000)	75 (4.2)	40 559 (8.0)	228.74 ± 327.89	70.95 (304.72)	
Medium 2 (70 001 to 100 000)	47 (2.6)	34 687 (6.8)	230.06 ± 261.15	155.49 (318.76)	
Large (> 100 000)	62 (3.5)	286 079 (56.2)	275.22 ± 463.23	144.89 (272.19)	
Large 1 (100 001 to 500 000)	51 (2.8)	113 407 (22.3)	274.27 ± 501.79	139.92 (235.02)	
Large 2 (> 500 000)	11 (0.7)	172 672 (33.9)	279.61 ± 223.50	201.81 (373.40)	

^a^ A case with ignored municipality

^b^ Kruskal–Wallis test; *SD* Standard deviation; *IQR* Interquartile range

### High-risk clusters are distributed throughout the Northeast region

**Fig. 4:**
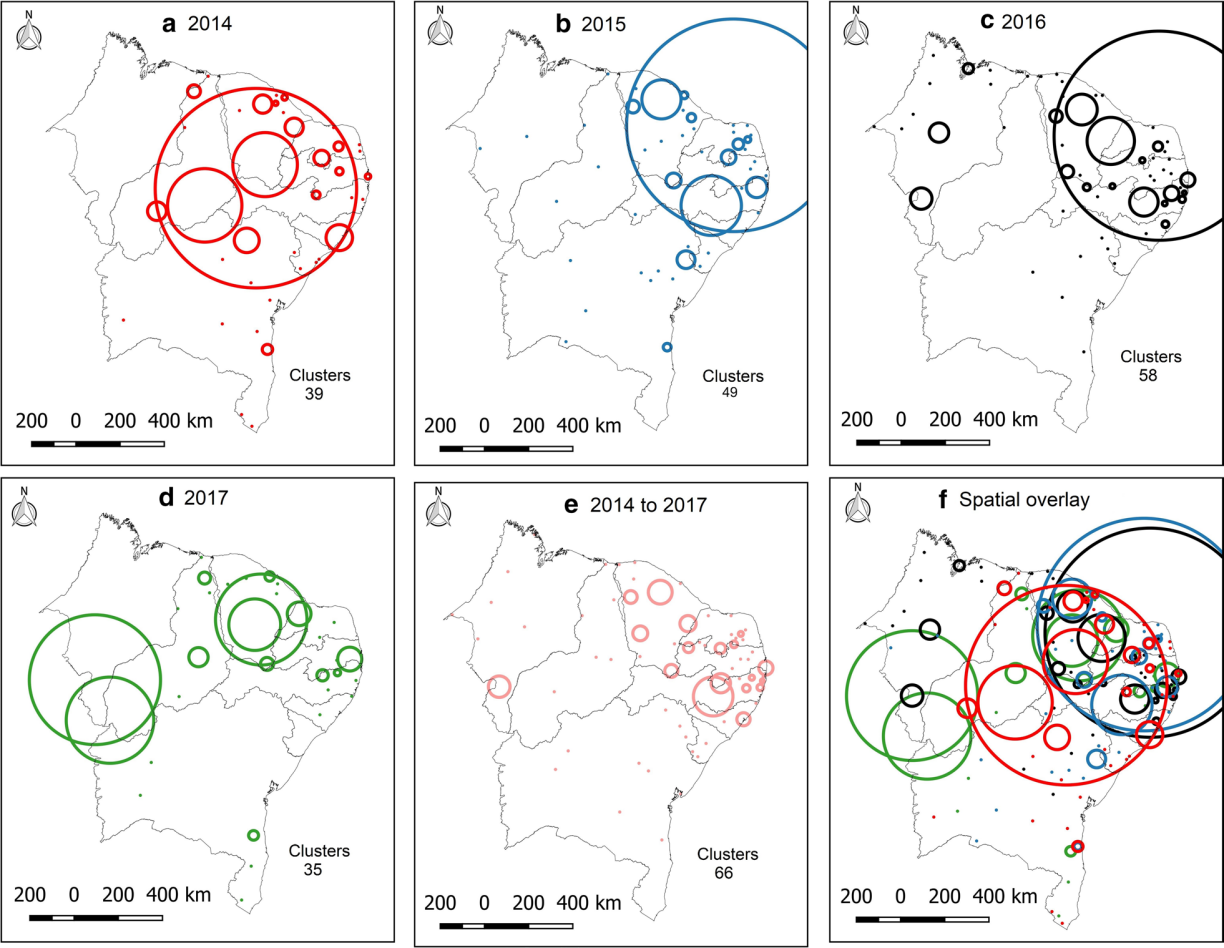
Statistics of spatial scanning of the incidence of dengue in Northeast Brazil. **a** 2014; **b** 2015; **c** 2016; **d** 2017; **e** 2014–2017; **f** Spatial overlay. Circles represent clusters of incidence

The spatial analysis stratified by year of the time series showed the displacement of areas at high risk of dengue in the Northeast. In 2014, there was a large cluster of risk in the region, except for part of Bahia and the entire state of Maranhão. In the following year (2015), this cluster moves to an axis that goes from the state of Alagoas to Ceará. In 2016, three clusters (with more than one municipality) appeared in Maranhão and, in 2017, the growth of risk areas in Ceará and the south of Maranhão, Piauí and western Bahia stood out. Considering the sum of cases in the period, 66 spatial clusters were identified (Fig. 4).

**Fig. 5:**
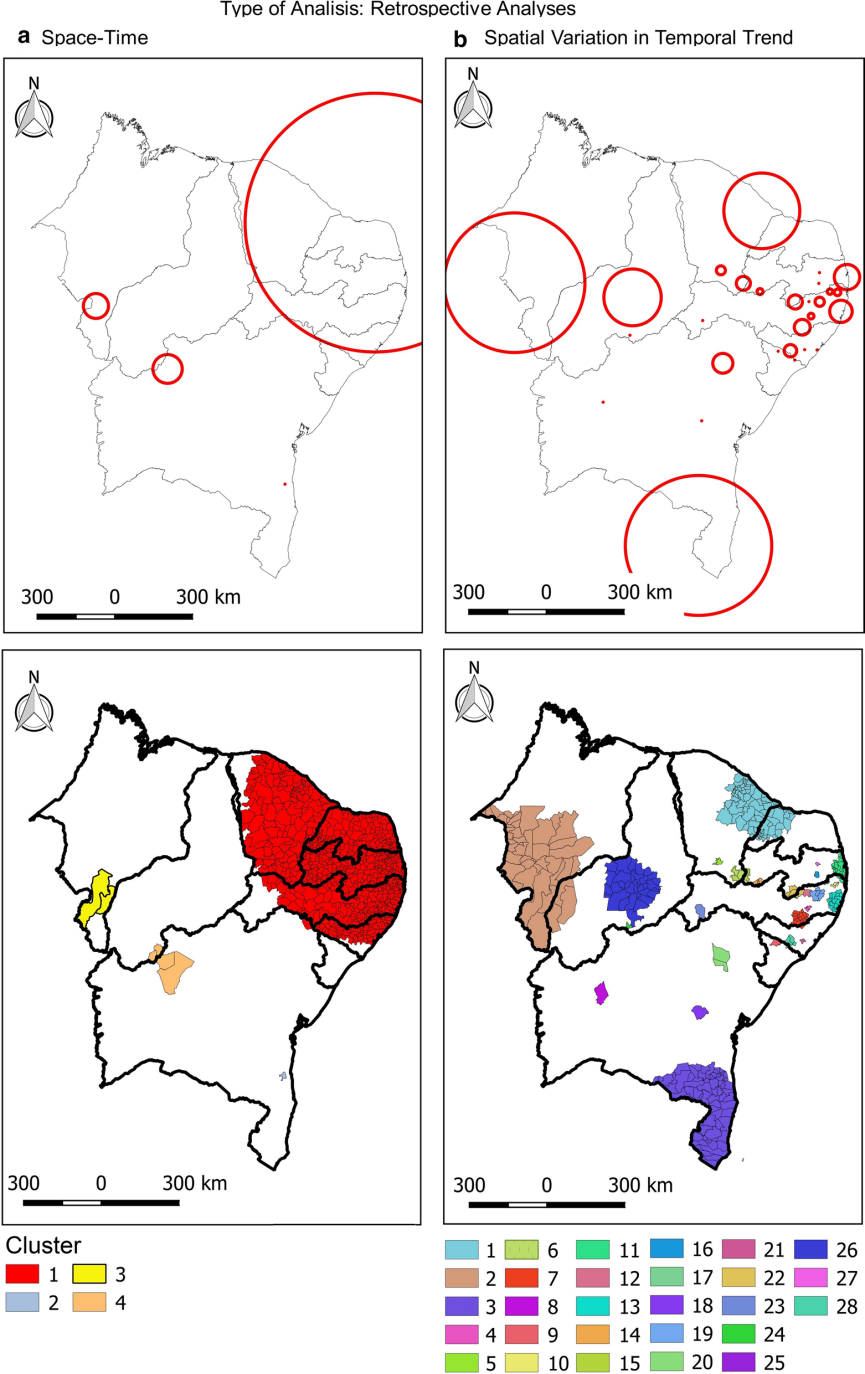
Retrospective analysis of space–time and spatial variation in the temporal trend of dengue incidence in Brazil Northeast, 2014–2017. **a** Space–time; **b** spatial variation in temporal trend. Circles represent spatial clusters in kilometers. Areas in color represent clusters in the level of municipalities

In the spatiotemporal analysis, four risk clusters were observed, involving 815 municipalities (45.45%) in the Northeast. Cluster 1 stood out, with 808 (45.06%) municipalities in the states of Alagoas, Pernambuco, Paraíba, Rio Grande do Norte, and Ceará, with an incidence rate of 524.3/100 000 and a relative risk of 4.07 (*P* < 0.001). Bahia, Maranhão and Piauí also presented risk areas in their territory (clusters 2, 3 and 4). In addition, the spatial variation in the temporal trend showed that all the states in the Northeast presented areas of risk, with emphasis on Pernambuco, with 10 clusters (Fig. 5 and Table 3).

**Table 3:** Retrospective analyses of space–time and spatial variation in temporal trend of Dengue incidence in Brazil Northeast, 2014–2017

a. Space–time								
Cluster	Time period	States	Radium (km)	Number of municipalities	Number of cases	Annual rate/100 000	Relative risk	*P-*value
1	2015–2016	Alagoas, Pernambuco, Paraíba, Rio Grande do Norte and Ceará	533.39	808	287 252	524.3	4.07	< 0.001
2	2015–2016	Bahia (Itabuna)	0.0	1	25 906	5882.8	27.56	< 0.001
3	2016–2017	Maranhão	51.35	2	2153	1049.8	4.69	< 0.001
4	2015–2015	Bahia and Piauí	59.38	4	1012	1092.7	4.87	< 0.001

b. Spatial variation in temporal trend								
Cluster	Inside time trend (%)	Localization	Radium (km)	Number of municipalities	Number of cases	Annual rate/100 000	Relative risk	*P-value*
1	10.53	Rio Grande do Norte and Ceará	155.93	82	101 814	466.3	2.35	0.001
2	84.89	Tocantins and Maranhão	287.79	41	8850	214.6	0.96	0.001
3	19.56	South of Bahia	288.43	65	38 094	434.6	2.01	0.001
4	2354.45	Agreste Pernambucano	11.76	2	662	351.2	1.57	0.001
5	192.11	South of Ceará	18.52	2	920	877.7	3.92	0.001
6	36.93	Ceará and Paraíba	28.82	6	5421	856.7	3.85	0.001
7	23.53	Pernambuco and Alagoas	32.32	14	6109	434.2	1.95	0.001
8	1365.01	West of Bahia	0.0	1	204	392.6	1.75	0.001
9	Inf	Sergipe	0.0	1	2	256.3	0.007	0.001
10	45.23	Zona da mata of Pernambuco	13.59	3	1626	319.6	1.43	0.001
11	3.14	East of Pernambuco and Paraíba	52.02	25	21 042	346.3	1.57	0.001
12	89.79	Agreste Pernambucano	0.0	1	358	181.4	0.81	0.001
13	2.87	East of Pernambuco	45.90	15	13 000	204.4	0.91	0.001
14	28.12	Sertão of Paraíba	12.32	3	1106	725.3	3.24	0.001
15	128.66	Sergipe	0.0	1	85	71.5	0.32	0.001
16	52.06	Paraíba	0.0	1	238	136.15	0.61	0.001
17	42.88	Pernambuco	11.19	3	273	119.9	0.53	0.001
18	17.80	Bahia	0.0	1	783	294.6	1.31	0.001
19	5.02	Pernambuco	18.94	5	2842	155.2	0.69	0.001
20	40.71	Bahia	40.75	2	242	67.9	0.30	0.001
21	28.15	Alagoas	0.0	1	385	383.7	1.71	0.001
22	4.28	Paraíba and Pernambuco	29.07	5	2543	1502.5	7.77	0.001
23	43.77	Pernambuco	0.0	1	157	157.7	0.70	0.001
24	Inf	Piauí	0.0	1	11	56.1	0.25	0.001
25	45.81	Alagoas	0.0	1	123	452.5	2.02	0.001
26	15.03	Piauí	117.04	30	488	40.2	0.18	0.001
27	19.66	Paraíba	0.0	1	327	248.4	1.11	0.001
28	25.48	Alagoas and Sergipe	25.48	4	722	213.0	0.95	0.001

*Inf* Infinite

### Social determinants can influence dengue incidence rate

All social indicators showed global spatial dependence. In the bivariate analysis, 9 indicators showed a spatial correlation with the dengue incidence rates (raw and smoothed) (Table 4). Among them, two showed a negative correlation (percentage of income from work and percentage of households with access to piped water) and seven showed a positive correlation (population density; percentage of people aged 6 to 14 who do not attend school; rate of illiteracy of individuals aged 18 or over; percentage of people aged 18 or over without complete elementary school and in an informal occupation; percentage of people aged 15 to 24 who do not study, do not work and have a per capita household income equal to or less than half the minimum wage (2010); percentage of households with access to electricity; percentage of mothers who are heads of household without elementary school and with minor children, in the total of mothers who are heads of households).

**Table 4:** Univariate and bivariate Moran statistics between raw and smoothed dengue incidence rates and social indicators. Northeast, Brazil, 2014–2017

Indicator	Univariate Moran’s *I* (*P*-value)	Bivariate Moran’s I (*P-*value)	
		Raw rate	Smoothed rate
Raw dengue incidence rate	0.3104 (*P* = 0.001)*	**–**	**–**
Smoothed dengue incidence rate	0.3229 (*P* = 0.001)*	**–**	**–**
a. Population			
% urban population	0.0377 (*P* = 0.02)*	0.0021 (*P* = 0.32)	0.0019 (*P* = 0.33)
Population density (inhab/km^2^)	0.1079 (*P* = 0.002)*	0.0134 (*P* = 0.04)*	0.0136 (*P* = 0.03)*
b. Education			
% of people aged 6 to 14 who do not attend school	0.1603 (*P* < 0.001)*	0.0159 (*P* = 0.01)*	0.0163 (*P* = 0.01)*
Illiteracy rate of individuals aged 18 or over	0.4016 (*P* < 0.001)*	0.0233 (*P* < 0.001)*	0.2318 (*P* = 0.004)*
% of people aged 18 or over without complete elementary education and in informal occupation	0.1600 (*P* < 0.001)*	0.0286 (*P* = 0.002)*	0.0293 (*P* = 0.002)*
c. Income			
Average income of the employed	0.2166 (*P* < 0.001)*	0.0087 (*P* = 0.09)	0.0087 (*P* = 0.09)
% of income from work	0.02783 (*P* < 0.001)*	0.0272 (*P* = 0.001)*	0.0275 (*P* = 0.001)*
% of people aged 15 to 24 who do not study, do not work and have a per capita household income equal to or less than half the minimum wage (2010)	0.1600 (*P* < 0.001)*	0.0286 (*P* = 0.001)*	0.0293 (*P* = 0.001)*
d. Housing			
% of households with access to piped water	0.2995 (*P* < 0.001)*	− 0.0356 (*P* = 0.005)*	− 0.0349 (*P* = 0.005)*
% of households with access to electricity	0.4378 (*P* < 0.001)*	0.0838 (*P* = 0.002)*	0.0841 (*P* = 0.002)*
e. Social vulnerability			
% of mothers who are heads of household without elementary school and with minor children, in the total of mothers who are heads of families	0.4378 (*P* < 0.001)*	0.0972 (*P* = 0.002)*	0.0978 (*P* = 0.002)*
% of vulnerable people who spend more than an hour to work among the employed population	0.3793 (*P* < 0.001)*	− 0.0031 (*P* = 0.31)	− 0.0035 (*P* = 0.29)

^*^Statistically significant (*P* < 0.05); %: Percentage

## Discussion

This study demonstrated the presence of spatial clusters in Northeastern Brazil, with risk areas distributed in all states, mainly in Ceará and Pernambuco states. A total of four risk clusters were observed, involving 815 municipalities (45.45%). We also provided evidence that socioeconomic factors such as population density, education level, income, housing and social vulnerabilities may contribute to dengue burden.

In the analyzed period, 509 261 cases of dengue were confirmed in the Northeast, an incidence rate of 224 cases per 100 000 inhabitants. This result is close to that found in a study carried out in Brazil between 1990 and 2017, which found an incidence rate for the northeast region of 246 cases per 100 000 inhabitants (Table 1) [7]. The semiarid climate, which is predominant in the Northeast, combined with poor sociodemographic conditions may contribute to the high rates of dengue in this region [28].

The year 2015 was responsible for the majority of cases registered in the period (43.2%) (Table 1). This year was marked by one of the largest dengue epidemics that Brazil has ever had, with 1 688 688 cases recorded, an incidence of 826 cases per 100 000 inhabitants, and co-circulation of the four DENV serotypes [7, 10]. On the other hand, in 2017 there was a drastic reduction in dengue incidence compared to previous years. The causes of this decline are still not fully understood [29] but may involve several factors including the existence of cross-immunity between Zika virus and dengue viruses [30, 31], and an overestimation of dengue notifications in 2015 and 2016 due to the co-circulation of Zika and chikungunya, which are arboviruses with similar symptoms [32].

The states of Ceará and Pernambuco contributed 53.41% of the total cases, with their capitals (Fortaleza and Recife, respectively), responsible for the highest both absolute numbers of cases and the highest incidence rate among all capitals in the region (Fig. 2 and Table 1). In line with our results, a recent study demonstrated that Ceará and Pernambuco ranked first among the Northeast states in number of fatal dengue cases in 30 years (1986–2015), reporting 506 and 277 cases, respectively [10].

Fortaleza and Recife have the highest population densities among the capitals of Northeastern Brazil (7786 inhabitants/km^2^ and 7037 inhabitants/km^2^, respectively) [33]. In these metropolises, the combination of a high number of inhabitants and a reduced geographical area, associated with specific social and environmental factors, favors the increase of the vector population and the interaction between it and the susceptible population [34]. Given this, local studies are also strongly recommended to understand the dynamics of dengue transmission in these cities.

The Bayesian model is an important approach to reduce random fluctuation caused by rare events, especially in municipalities with small populations and underreporting cases [25]. In the present study, the application of this model reduced the number of municipalities without reported cases of dengue from 134 to 3. In the Moran Map, 107 municipalities were classified as priority, mainly those located between the states of Ceará and Alagoas (Fig. 2). These municipalities are considered priority, since they are located in the Q1 quadrant of the Moran map, which means that both they and their neighbors have a high dengue incidence rate.

Indeed, underreporting is a major challenge for disease surveillance, especially in dengue. A study carried out in Salvador, Northeastern Brazil, showed that for every 20 dengue patients identified, only about one had been reported to the surveillance system as having dengue. During periods of low dengue transmission, only about one in 40 dengue cases identified was reported [35]. Therefore, the Bayesian approach model can be applied as an alternative to the traditional models for the study of spatial analysis for the definition of strategic areas for the establishment of control and prevention actions.

The spatial analysis identified high-risk dengue clusters in the Northeast of Brazil in the studied years. Initially, in 2014, dengue cases were concentrated in the center of the Northeastern region, covering most states. In 2015, these clusters moved to the east coast, concentrating mainly between the states of Ceará and Alagoas. In 2016, clusters appeared in some municipalities in Maranhão and in 2017 there is an expansion of dengue, mainly in southern Maranhão and Piauí, and western Bahia. When performing the spatiotemporal analysis, the presence of high-risk clusters in these regions is evident (Fig. 5). Our results are consistent with previous findings that dengue is spatially correlated with clusters [36–39]. It demonstrates the wide spatial spread of dengue and the exposure of a significant portion of the population in the Northeast Brazil.

In the present study, both population size and population density were correlated with an increased incidence of dengue. Municipalities with more than 100 thousand inhabitants had an incidence rate almost 2 times higher than those with less than 50 thousand inhabitants (Table 2). The relationship between population growth and dengue incidence has been demonstrated previously [40–42]. DENV has fully adapted to a human-*Ae. aegypti*-human transmission cycle in the large urban centers of the tropics, where crowded human populations live in intimate association with equally large mosquito populations [40]. In addition, more populated environments favor the vector proliferation and multiple feeding, thus amplifying DENV transmission dynamics [41, 43].

Rapid and unplanned urbanization with poor sanitary conditions, deterioration of the public health infrastructure, decreased access to health care and inadequate vector-control efforts contribute to the increase of dengue burden [4]. In this study, we demonstrated that social determinants showed spatial correlation with dengue incidence. Access to piped water was negatively correlated with dengue incidence (Table 4). The absence of piped water leads the population to keep water in containers, normally discovered, facilitating the reproduction of mosquitoes and increasing the risk of dengue [40, 44, 45]. Therefore, improving piped water infrastructure may reduce dengue occurrence. Other studies also found that access to piped water and water supply interruptions were important risk factors for the presence of *Ae. aegypti* and dengue [44, 46, 47]. On the other hand, access to electricity was associated with higher dengue incidence in the present study. This association is not expected, since low access to electricity could be associated with more precarious living conditions. However, the larger access to electricity in urban areas that also have larger social inequalities could explain, at least in part, the association between electricity and dengue fever incidence in our study.

We observed a spatial correlation between income and dengue incidence. Maccormack-Gelles et al. [18] reported that, in Fortaleza (Brazilian Northeast), a USD 178.58 (USD 1 = BRL 5.60) increase in average annual bairro household income was associated with reduced dengue incidence by more than 10% [18]. In Brazil, inadequate garbage disposal and income were the most significant factors related to the incidence of dengue [42, 48], and lower socio-economic status (within a slum society) increased the risk of dengue [36, 49].

Educational indicators associated with illiteracy and low education level showed direct correlation with dengue incidence in this study (Table 4). The relationship between education level and dengue risk presents divergent data in the literature. Siqueira et al. [50] demonstrated that the risk of DENV infection was associated with older age, low education, and low income, in a household survey conducted in Goiania, Central-Western Brazil [50]. In Fortaleza, Brazil, male literacy was associated with increased dengue incidence rates while female literacy was correlated with lower rates [18]. A study in the city of Rio de Janeiro, Brazil, found a positive association between the adult literacy rate and lack of access to piped water with the risk of dengue [51]. In Indonesia, education level was an important risk factor associated with dengue. According to the study, populations with high levels of education and employment are more likely to seek healthcare when infected with dengue than poor populations [52]. The difference in the results found in the other studies and in ours may be explained by the difference in the chosen indicators, since the low level of education is generally associated with more unfavorable socio-economic conditions.

Regarding social vulnerability indicators, we found a positive association between dengue incidence and percentage of mothers who are heads of household without elementary school and with minor children (Table 4). In Mexico, households where the mother did not complete primary school, were two times more likely to have more larval breeding containers. According to the authors, although housewives knew of the presence of *Ae. aegypti* larvae in their houses, they were unaware of their potentiality as biting mosquitoes, and less of their potentiality as dengue vectors [53]. The relationship between the low level of education of mothers and the risk for diseases has been demonstrated in previous studies [54–57].

This context shows that public policies aimed at tackling dengue must be broad and encompass two main groups of actions: The first should be directed to actions related to disease surveillance, such as vector monitoring and control, expansion of human resources, notification and investigation of suspected cases, identification and monitoring of risk areas, and management of environmental conditions. The second group of measures should focus on the population's living conditions, such as access to deceived water, education and income. Otherwise, without the combined adoption of these two groups of measures, it is unlikely that dengue containment strategies will achieve the expected results.

Our study has some limitations worth noting. The dengue surveillance system in Brazil is not completely accurate. Underreporting may occur in cases where infected individuals with mild or asymptomatic symptoms do not seek medical assistance, or symptomatic individuals who are misdiagnosed with another febrile illness. Overestimation may also occur due to other vector-borne diseases with similar symptoms, like Zika or chikungunya. Another issue is incorrect records, with incomplete data and lack of reporting. The lack of information on local actions that can impact the incidence of the disease is also another limitation. The use of a small-time series (only 4 years), possibly compromised the trend analysis. Lastly, information about the social indicators were only available for the year of 2010.

## Conclusions

We demonstrate the dynamics of DENV infection in Northeastern Brazil in a time series using spatial analysis tools. The spatial distribution of the disease is considerably heterogeneous and there are areas of high risk of transmission in the region.

A total of nine social indicators were identified as social determinants of dengue in Northeastern region. These indicators, in turn, are related to the different social aspects: population density, education, income, housing, and social vulnerability. Finally, the results presented in the present study can provide subsidies for decision-making in public health policies aiming at the reduction and greater control of dengue cases.

## Data Availability

The datasets analyzed during the current study are available in the National Notifiable Diseases Information System—SINAN (https://datasus.saude.gov.br/) and the Brazilian Institute of Geography and Statistics (IBGE) (https://sidra.ibge.gov.br/home/pmc/brasil).

## Abbreviations

APC: Annual percent change
DENV: Dengue virus
IBGE: Brazilian Institute of Geography and Statistics
WHO: World Health Organization

## References

[1] Bhatt S, Gething PW, Brady OJ, Messina JP, Farlow AW, Moyes CL (2013). The global distribution and burden of dengue. Nature.

[2] Guzman MG, Gubler DJ, Izquierdo A, Martinez E, Halstead SB (2016). Dengue infection. Nat Rev Dis Primers.

[3] Guzman MG, Harris E (2015). Dengue. Lancet.

[4] San Martín JL, Brathwaite O, Zambrano B, Solórzano JO, Bouckenooghe A, Dayan GH (2010). The epidemiology of dengue in the Americas over the last three decades: a worrisome reality. Am J Trop Med Hyg.

[5] Teurlai M, Menkès CE, Cavarero V, Degallier N, Descloux E, Grangeon J-P (2015). Socio-economic and climate factors associated with dengue fever spatial heterogeneity: a worked example in New Caledonia. PLoS Negl Trop Dis.

[6] PAHO—Pan American Health Organization. Health information platform for the Americas (PLISA, PAHO/WHO). https://www.paho.org/data/index.php/en/. Accessed 20 May 2020.

[7] Andrioli DC, Busato MA, Lutinski JA (2020). Spatial and temporal distribution of dengue in Brazil, 1990–2017. PLoS ONE.

[8] Araújo VEMd, Bezerra JMT, Amâncio FF, Passos VMdA, Carneiro M (2017). Increase in the burden of dengue in Brazil and federated units, 2000 and 2015: analysis of the Global Burden of Disease Study 2015. Rev Bras Epidemiol.

[9] Martins-Melo FR, Carneiro M, Ramos AN, Heukelbach J, Ribeiro ALP, Werneck GL (2018). The burden of neglected tropical diseases in Brazil, 1990–2016: a subnational analysis from the Global Burden of Disease Study 2016. PLoS Negl Trop Dis.

[10] Nunes PCG, Daumas RP, Sánchez-Arcila JC, Nogueira RMR, Horta MAP, dos Santos FB (2019). 30 years of fatal dengue cases in Brazil: a review. BMC Public Health.

[11] Burattini MN, Lopez LF, Coutinho FA, Siqueira-Jr JB, Homsani S, Sarti E (2016). Age and regional differences in clinical presentation and risk of hospitalization for dengue in Brazil, 2000–2014. Clinics.

[12] IPEA. Desenvolvimento humano nas macrorregiões brasileiras: 2016. PNUD, IPEA, FJP. 2016. https://repositorio.ipea.gov.br/bitstream/11058/6217/1/Desenvolvimento%20humano%20nas%20macrorregi%C3%B5es%20brasileiras.pdf. Accessed 20 May 2020.

[13] Instituto Brasileiro de Geografia e Estatística (IBGE). Síntese de indicadores sociais: uma análise das condições de vida da população brasileira: 2017. IBGE; 2017.

[14] Instituto Brasileiro de Geografia e Estatística (IBGE). Pesquisa Nacional por Amostra de Domicílios contínua 2016: outras formas de trabalho. IBGE; 2017.

[15] Costa AIPd, Natal D (1998). Distribuição espacial da dengue e determinantes socioeconômicos em localidade urbana no Sudeste do Brasil. Rev Saude Publica.

[16] Mendonça FdA, Souza AV, Dutra DdA (2009). Saúde pública, urbanização e dengue no Brasil. Soc Nat.

[17] Braga C, Luna CF, Martelli CM, De Souza WV, Cordeiro MT, Alexander N (2010). Seroprevalence and risk factors for dengue infection in socio-economically distinct areas of Recife, Brazil. Acta Trop.

[18] MacCormack-Gelles B, Neto ASL, Sousa GS, Nascimento OJ, Machado MM, Wilson ME (2018). Epidemiological characteristics and determinants of dengue transmission during epidemic and non-epidemic years in Fortaleza, Brazil: 2011–2015. PLoS Negl Trop Dis.

[19] Caprarelli G, Fletcher S (2014). A brief review of spatial analysis concepts and tools used for mapping, containment and risk modelling of infectious diseases and other illnesses. Parasitology.

[20] Santos DNd, da Silva VdP, Sousa FdA, Silva RA (2010). Estudo de alguns cenários climáticos para o Nordeste do Brasil. Rev Bras Eng Agric Amb.

[21] World Health Organization (2009). Dengue: guidelines for diagnosis, treatment, prevention and control.

[22] National Notifiable Diseases Information System (SINAN). https://datasus.saude.gov.br/. Accessed 20 May 2020.

[23] Instituto Brasileiro de Geogragia e Estatística (IBGE). https://sidra.ibge.gov.br/home/pmc/brasil. Accessed 20 May 2020.

[24] Kim HJ, Fay MP, Feuer EJ, Midthune DN (2000). Permutation tests for joinpoint regression with applications to cancer rates. Stat Med.

[25] Souza WV, Barcellos CC, Brito AM, Carvalho MS, Cruz OG, Albuquerque MFM (2001). Aplicação de modelo bayesiano empírico na análise espacial da ocorrência de hanseníase. Rev Saude Publica.

[26] Druck S, Carvalho MS, Câmara G, Monteiro AVM (2004). Análise espacial de dados geográficos.

[27] Kulldorff M (1997). A spatial scan statistic. Commun Stat Theory Methods.

[28] Rodrigues NCP, Lino VTS, Daumas RP, de Noronha Andrade MK, O’Dwyer G, Monteiro DLM (2016). Temporal and spatial evolution of dengue incidence in Brazil, 2001–2012. PLoS ONE.

[29] Lopes TRR, Silva CS, Pastor AF, Silva Júnior JVJ (2018). Dengue in Brazil in 2017: what happened?. Rev Inst Med Trop Sao Paulo.

[30] Ribeiro GS, Kikuti M, Tauro LB, Nascimento LCJ, Cardoso CW, Campos GS (2018). Does immunity after Zika virus infection cross-protect against dengue?. Lancet Glob Health.

[31] Rodriguez-Barraquer I, Costa F, Nascimento EJ, Nery N, Castanha PM, Sacramento GA (2019). Impact of preexisting dengue immunity on Zika virus emergence in a dengue endemic region. Science.

[32] Perez F, Llau A, Gutierrez G, Bezerra H, Coelho G, Ault S (2019). The decline of dengue in the Americas in 2017: discussion of multiple hypotheses. Trop Med Int Health.

[33] Instituto Brasileiro de Geografia e Estatística (IBGE). www.ibge.gov.br/home/estatistica/populacao/censo2010. Accessed 20 May 2020.

[34] Barcellos C, Lowe R (2014). Expansion of the dengue transmission area in Brazil: the role of climate and cities. Trop Med Int Health.

[35] Silva MM, Rodrigues MS, Paploski IA, Kikuti M, Kasper AM, Cruz JS (2016). Accuracy of dengue reporting by national surveillance system, Brazil. Emerg Infect Dis.

[36] Aswi A, Cramb S, Moraga P, Mengersen K (2018). Bayesian spatial and spatio-temporal approaches to modelling dengue fever: a systematic review. Epidemiol Infect.

[37] Stewart-Ibarra AM, Muñoz ÁG, Ryan SJ, Ayala EB, Borbor-Cordova MJ, Finkelstein JL (2014). Spatiotemporal clustering, climate periodicity, and social-ecological risk factors for dengue during an outbreak in Machala, Ecuador, in 2010. BMC Infect Dis.

[38] Almeida ASd, Medronho RdA, Valencia LIO (2009). Spatial analysis of dengue and the socioeconomic context of the city of Rio de Janeiro (Southeastern Brazil). Rev Saude Publica.

[39] Dalvi A, Braga J (2019). Spatial diffusion of the 2015–2016 Zika, dengue and chikungunya epidemics in Rio de Janeiro Municipality, Brazil. Epidemiol Infect.

[40] Gubler DJ (2011). Dengue, urbanization and globalization: the unholy trinity of the 21st century. Trop Med Health.

[41] Struchiner CJ, Rocklöv J, Wilder-Smith A, Massad E (2015). Increasing dengue incidence in Singapore over the past 40 years: population growth, climate and mobility. PLoS ONE.

[42] Honorato T, Lapa PPA, Sales CMM, Reis-Santos B, Tristão-Sá R, Bertolde AI (2014). Spatial analysis of distribution of dengue cases in Espírito Santo, Brazil, in 2010: use of Bayesian model. Rev Bras Epidemiol.

[43] Scott TW, Clark GG, Lorenz LH, Amerasinghe PH, Reiter P, Edman JD (1993). Detection of multiple blood feeding in *Aedes aegypti* (Diptera: Culicidae) during a single gonotrophic cycle using a histologic technique. J Med Entomol.

[44] Stewart Ibarra AM, Ryan SJ, Beltrán E, Mejía R, Silva M, Muñoz ÁG (2013). Dengue vector dynamics (*Aedes aegypti*) influenced by climate and social factors in Ecuador: implications for targeted control. PLoS ONE.

[45] Guo C, Zhou Z, Wen Z, Liu Y, Zeng C, Xiao D (2017). Global epidemiology of dengue outbreaks in 1990–2015: a systematic review and meta-analysis. Front Cell Infect Microbiol.

[46] Caprara A, Lima JWdO, Marinho ACP, Calvasina PG, Landim LP, Sommerfeld J (2009). Irregular water supply, household usage and dengue: a bio-social study in the Brazilian Northeast. Cad Saude Publica.

[47] Ryan SJ, Lippi CA, Nightingale R, Hamerlinck G, Borbor-Cordova MJ, Cruz B (2019). Socio-ecological factors associated with dengue risk and *Aedes aegypti *presence in the Galápagos Islands, Ecuador. Int J Environ Res Public Health.

[48] Barcellos C, Pustai AK, Weber MA, Brito MRV (2005). Identificação de locais com potencial de transmissão de dengue em Porto Alegre através de técnicas de geoprocessamento. Rev Soc Bras Med Trop.

[49] Kikuti M, Cunha GM, Paploski IA, Kasper AM, Silva MM, Tavares AS (2015). Spatial distribution of dengue in a Brazilian urban slum setting: role of socioeconomic gradient in disease risk. PLoS Negl Trop Dis.

[50] Siqueira JB, Martelli CM, Maciel IJ, Oliveira RM, Ribeiro MG, Amorim FP (2004). Household survey of dengue infection in central Brazil: spatial point pattern analysis and risk factors assessment. Am J Trop Med Hyg.

[51] Ferreira GS, Schmidt AM (2006). Spatial modelling of the relative risk of dengue fever in Rio de Janeiro for the epidemic period between 2001 and 2002. Braz J Probab Stat.

[52] Wijayanti SP, Porphyre T, Chase-Topping M, Rainey SM, McFarlane M, Schnettler E (2016). The importance of socio-economic versus environmental risk factors for reported dengue cases in Java, Indonesia. PLoS Negl Trop Dis.

[53] Danis-Lozano R, Rodríguez MH, Hernández-Avila M (2002). Gender-related family head schooling and Aedes aegypti larval breeding risk in Southern Mexico. Salud Publica Mex.

[54] Vlassoff C, Bonilla E (1994). Gender-related differences in the impact of tropical diseases on women: what do we know?. J Biosoc Sci.

[55] Marsh V, Mutemi W, Some E, Haaland A, Snow R (1996). Evaluating the community education programme of an insecticide-treated bed net trial on the Kenyan coast. Health Policy Plan.

[56] Cleland JG, Van Ginneken JK (1988). Maternal education and child survival in developing countries: the search for pathways of influence. Soc Sci Med.

[57] Njau JD, Stephenson R, Menon MP, Kachur SP, McFarland DA (2014). Investigating the important correlates of maternal education and childhood malaria infections. Am J Trop Med Hyg.

